# Effects of Exercise-Based Telerehabilitation for Knee Osteoarthritis: A Systematic Review and a Study Protocol

**DOI:** 10.3390/bioengineering13020136

**Published:** 2026-01-24

**Authors:** Giacomo Farì, Francesco Quarta, Federica Bressi, Raffaele La Russa, Teresa Paolucci, Andrea Bernetti

**Affiliations:** 1 Department of Experimental Medicine (Di.Me.S), University of Salento, Piazza Tancredi, 7, 73100 Lecce, Italy; andrea.bernetti@unisalento.it; 2 Department of Biological and Environmental Science and Technologies (Di.S.Te.B.A.), University of Salento, Piazza Tancredi, 7, 73100 Lecce, Italy; francesco.quarta6@studenti.unisalento.it; 3 Physical Medicine and Rehabilitation Unit, Campus Bio-Medico University Polyclinic Foundation of Rome, Via Álvaro del Portillo, 200, 00128 Rome, Italy; f.bressi@policlinicocampus.it; 4 Department of Clinical Medicine, Public Health, Life Sciences, and Environmental Sciences, University of L’Aquila, Piazza Santa Margherita, 2, 67100 L’Aquila, Italy; raffaele.larussa@univaq.it; 5 Department of Medical, Oral and Biotechnological Sciences (DSMOB), G. D’Annunzio University of Chieti-Pescara, Via dei Vestini, 31, 66100 Chieti, Italy; teresapaolucci@hotmail.com; 6 Interdepartmental University Program of Physical and Rehabilitation Medicine, “V. Fazzi” Hospital, ASL Lecce, 73100 Lecce, Italy

**Keywords:** osteoarthritis, knee, telemedicine, digital medicine, rehabilitation, systematic review

## Abstract

Background: Knee osteoarthritis causes considerable pain and disability. Telerehabilitation has emerged as a promising treatment option, especially after the Coronavirus Disease 2019 pandemic, but it still faces challenges regarding solid scientific evidence about its multiple benefits. This systematic review aimed to analyze the reported beneficial effects of telerehabilitation based on therapeutic exercise for the management of knee osteoarthritis. Methodsː PubMed, PEDro, Web of Science and Cochrane Library databases were used to identify eligible studies. This review followed the PRISMA guidelines and was registered at PROSPERO (n° CRD42024579836). The selected studies underwent a qualitative assessment using the Modified Jadad Score. Results: Ten studies, including a total of 1354 participants, were included. From the selected studies, a wide variety of outcome measures emerged to evaluate the efficacy of telerehabilitation in the relief of pain and its clinical consequences. Seven studies specifically assessed pain, with four showing significant improvements in pain reduction in the intervention group compared with the control group. Telerehabilitation was found to be more effective or non-inferior to traditional rehabilitation in relieving pain, as reported across various pain scales. Limitations include the heterogeneity of interventions, the exclusion of non-recent studies, and the exclusive focus on therapeutic exercise. Conclusionsː The results of this systematic review suggest that telerehabilitation provides pain relief, improves physical function, and enhances quality of life, while preliminary evidence indicates potential cost-related advantages. However, some studies did not find TR to be superior to control interventions, highlighting mixed evidence. Additional high-quality studies are required to better support this promising rehabilitation approach.

## 1. Introduction

Knee osteoarthritis (KOA) is one of the most common forms of osteoarthritis, and its incidence is expected to increase in the coming decades [1]. Aging and obesity, particularly in industrialized countries, are major contributing factors to its rising prevalence [2]. KOA is a joint disease characterized by a chronic course, leading to progressive damage to the articular environment and resulting in severe knee pain and motor disability [3,4]. The challenge of treating KOA remains highly relevant due to its substantial public and private healthcare costs [5].

Telerehabilitation (TR) is an emerging modality in the field of telemedicine and e-health that applies telecommunication technologies to deliver rehabilitation services, with wearable sensors and remote monitoring playing a key role [6]. TR has rapidly expanded over the last two decades; however, starting in 2020, the Coronavirus Disease 2019 (COVID-19) pandemic significantly accelerated its clinical applications [7]. Recent studies have highlighted the potential of next-generation technologies, including TR, to enhance clinical practice and personalize care, particularly for conditions such as KOA [8]. Despite existing limitations [9], TR has been shown to be a promising intervention, especially in terms of pain relief and improvements in joint function, both for individuals with KOA [10] and for those undergoing knee replacement [11]. TR may also represent an opportunity to reduce the high costs associated with traditional rehabilitation, as it does not require large spaces and can also be delivered asynchronously. Moreover, TR may ensure access to adequate therapies regardless of geographic location or local rehabilitation service availability [12].

Nonetheless, TR has not yet achieved full implementation and still lacks robust supporting evidence. The existing evidence specifically focused on TR in KOA, although valuable, remains limited and often addresses only selected potential benefits. In addition, there is considerable heterogeneity in how TR is defined, with approaches ranging widely in terms of rehabilitation interventions and comparison groups, which makes it challenging to define its real effectiveness [13].

Therefore, the aim of this systematic review is to analyze the reported beneficial effects of TR based on therapeutic exercise delivered through different eHealth and digital therapy modalities, in comparison with traditional KOA rehabilitation.

## 2. Materials and Methods

### 2.1. Data Sources, Search Strategy and Study Selection

This review adhered to the Preferred Reporting Items for Systematic Reviews and Meta-Analyses (PRISMA) statement guidelines [14]. The protocol was registered in PROSPERO (n° CRD42024579836).

The scientific articles were identified through the PubMed, PEDro, Web of Science, and Cochrane Library databases, using the Medical Subject Headings (MeSH) terms when applicable. The following Boolean search syntax was applied: “(telerehabilitation OR telemedicine OR telehealth OR “tele health” OR “tele rehabilitation” OR “tele medicine” OR eHealth OR digital* OR mobile OR smartphone OR app OR virtual*) AND (“knee osteoarthritis” OR “knee arthrosis” OR gonarthrosis) AND (exercise OR “therapeutic exercise” OR “exercise-based rehabilitation” OR physiotherapy OR “physical therapy”)”.

The following filters were activated—text availability: full text; article type: clinical trial, randomized controlled trial; species: humans; article language: English; age: adults; period: last ten years (1 January 2014 to 30 June 2024). References of the included articles were manually examined for additional relevant publications. The inclusion criteria were the following: clinical trials involving adults (≥18 years) with KOA receiving therapeutic exercise-based TR delivered via any technological device and compared with traditional exercise-based rehabilitation or usual care. The exclusion criteria were the following: conference papers, study protocols, case reports, case series, studies describing a digital intervention delivered only by telephone calls or by gaming strategies, reviews and meta-analyses; trials involving TR for participants who had undergone knee surgery for KOA; trials focusing on TR for KOA related to other diseases, such as obesity.

### 2.2. Data Management, Data Extraction and Outcome Measures

Four investigators (G.F., F.Q., R.L.R., and T.P.) independently and carefully assessed each title, abstract, and full-text article to identify eligible studies. Disagreements were resolved by consensus by asking two other experienced investigators (A.B. and F.B.). All authors agreed on the final inclusion list.

A data extraction form was created in Microsoft Excel. Data extraction was independently performed by two authors (G.F. and F.Q.). Any discrepancies were resolved through discussion or, when necessary, consultation with a third reviewer (A.B.). Data were extracted regarding study characteristics (author, year, country, study design), population characteristics (health condition, sample size, gender, age) and intervention characteristics (type of exercise-based TR intervention, additional intervention, technology used, outcome measurements and key findings). Considering the heterogeneity of study designs, interventions, and outcome measures, a quantitative meta-analysis was not feasible. Therefore, findings were synthesized narratively [15], summarizing the direction of effect for each outcome across studies. All the outcome measures reported in the selected studies were collected. They included the following areas of interest: (1) pain; (2) physical function; (3) strength; (4) quality of life; (5) physical activity (PA) levels; (6) participants’ satisfaction; (7) cost-effectiveness. They were all extensively described in the results section. These are described in the Section 3.

### 2.3. Quality Assessment

The selected studies were qualitatively assessed using the Modified Oxford Quality Scoring System, which is also referred to as the Modified Jadad Score [16]. This five-item scale evaluates randomization and concealment of treatment allocation groups, withdrawals and dropout rates, adherence to inclusion and exclusion criteria, and the clarity of statistical methods reporting within the studies under analysis. The Modified Jadad score ranges from 0 to 5, and each question has a dichotomic answer (yes—1 point; no—0 points). Each aspect was assessed independently by the two investigators mentioned earlier. Studies scoring > 3 points were considered high quality; those scoring 2–3 were considered moderate quality, and those scoring < 2 points were considered low quality.

### 2.4. Risk of Bias Assessment

The risk of bias across the included studies was assessed using the five domains of the Cochrane Risk of Bias 2 (RoB 2) tool [17]: (1) bias arising from the randomization process; (2) bias due to deviations from intended interventions; (3) bias due to missing outcome data; (4) bias in measurement of the outcome; (5) bias in selection of the reported result. Each domain was judged as “low risk of bias” (“green”), “high risk of bias” (“red”), or “some concerns” (“yellow”). Results were summarized as percentages and visualized using the Risk of Bias visualization tool [18].

The RoB 2 assessment provides a detailed, domain-specific evaluation of potential biases, complementing the overall methodological quality rating provided by the Modified Jadad Score.

### 2.5. Dealing with Missing Data

Missing data were handled according to their reporting in the original studies. Available data were analyzed as presented, and no additional imputation or statistical adjustments were applied.

### 2.6. Possible Risks for Heterogeneity

Potential sources of heterogeneity may include differences in study design, statistical methods, participant populations, and clinical interventions.

## 3. Results

### 3.1. Identification of the Studies

**Figure 1:**
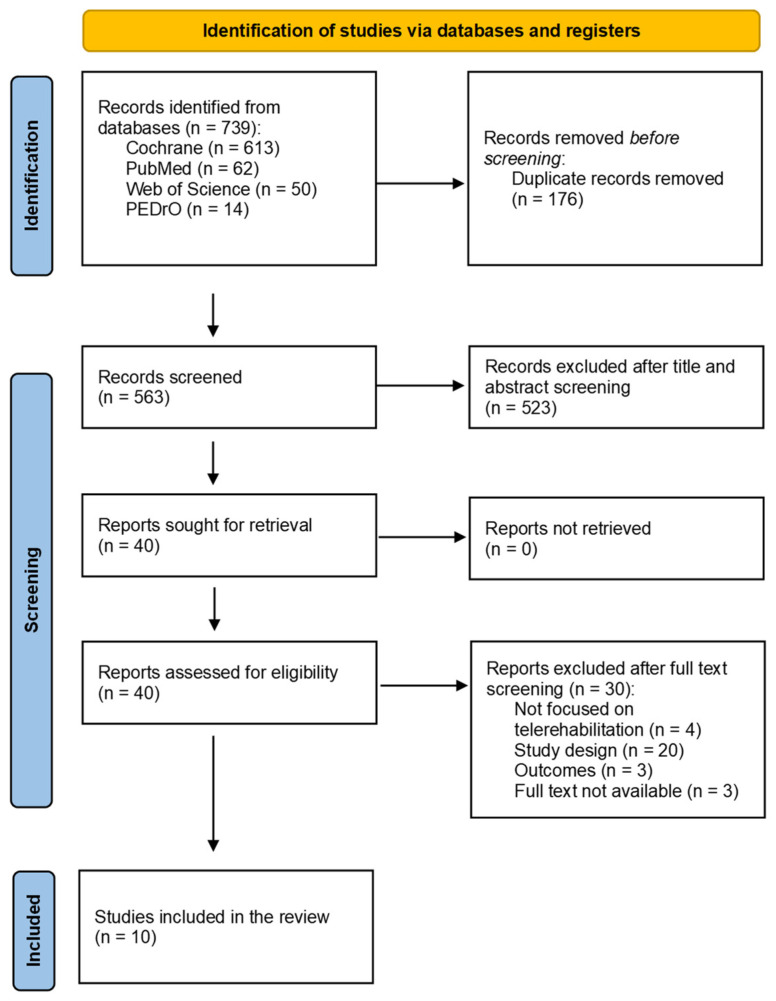
PRISMA Flow Diagram of Study Selection Process.

The studies were identified through a search in four databases (PubMed, PEDro, Web of Science and the Cochrane Library databases). At the end of the selection process, 739 articles were extracted, of which 613 were from Cochrane, 62 from PubMed, 50 from Web of Science, and 14 from PEDro. Then, duplicates were eliminated (*n* = 176). After title and abstract screening, 523 articles were excluded. The full text of the remaining 40 studies was assessed for eligibility. Thirty articles were excluded after full-text screening for the following reasons: the study was not focused on rehabilitation (*n* = 4), the study design did not meet the inclusion criteria (*n* = 20), the reported outcomes were not relevant (*n* = 3), or the full text was not available (*n* = 3). Finally, 10 research articles were included [19,20,21,22,23,24,25,26,27,28] (Figure 1).

### 3.2. Studies Characteristics

Characteristics of the included studies are presented in Table 1.

### 3.3. Results Presentation

All the selected studies were randomized controlled trials. Overall, 1354 participants were included in this review. Sample sizes ranged from 40 to 394 participants. Across studies, most participants were female, with proportions ranging from 26% to 100%. The study population primarily consisted of middle-aged and older adults, with mean ages ranging from 46 to 63.8 years.

The selected studies reported a wide range of technological solutions, devices, and delivery methods used to provide TR. Apps were the most frequently adopted technological solution (*n* = 9/10; 90%), either alone [19,20,21,22,23,25,27,28] or in combination with video recordings delivered via DVD [24]. In contrast, the study conducted by Odole et al. used mobile phones to deliver exercise instructions [26]. Specifically, two studies implemented e-Exercise in their research [21,22], two studies used WhatsApp [24,27], and other studies used various apps, including Join2Move [19], My Dear Knee [20], Hinge Health [23], Love-Your-Knee [25], and Zoom [28]. Additionally, the study by Mecklenburg et al. integrated Bluetooth sensors in combination with the Hinge Health app [23].

In most of the selected studies (9/10), the therapy sessions were conducted in an unsupervised format [19,20,21,22,23,24,25,26,27]. Notably, in two of these studies, unsupervised sessions were supplemented by approximately five face-to-face therapy sessions [21,22]. Only one study, conducted by Hinman et al., delivered therapy sessions in a supervised format via videoconference [28].

The duration of the interventions ranged from 4 to 14 weeks, with a predominance of 12-week programs (*n* = 5/10, 50%) [19,21,23,24,25,26,27,28].

Follow-up assessment time points varied considerably across studies. Four studies assessed outcomes only at the end of the intervention [19,23,25,27]. Two studies assessed the outcomes at intermediate time points: Alasfour et al. evaluated participants at 3 and 6 weeks (end of intervention) [20], and Odole et al. evaluated them at 2, 4 and 6 weeks (end of intervention) [26]. Four studies included post-intervention follow-up assessments. Specifically, Kloek et al., Kloek, and Bossen et al. evaluated the outcomes at the end of the intervention (third month) and nine months later [21,22]. Aily et al. conducted evaluations at two time points, after 14 weeks (end of intervention) and three months later [24]. Finally, Hinman et al. evaluated the effects at the end of the intervention (3 months) and six months later [28].

A wide variety of intervention types were used. Strength exercises were part of seven out of ten studies [19,20,21,22,25,27,28]. In most cases, strength training was part of a multicomponent exercise program including activities such as walking, swimming, or cycling [19,21,22], stretching [25,27], stability exercises [21,22,27], and core stability or postural exercises [19]. Conversely, Alasfour et al. designed an intervention program based exclusively on lower-limb strength exercises [20].

Educational programs or information modules related to KOA were also included in half of the selected studies [19,21,22,23,28]. One study structured its intervention around a training circuit composed of different therapeutic exercises [24].

**Table 1:** Summary characteristics of the selected studies. Regarding follow-up detection times, the number of weeks/months from the beginning of the therapy considered as intervention is reported.

Title; Authors; Year	Sample	Technology	Intervention Group	Control Group	Follow-Up Detection Times	Outcomes Measures and Evaluation Tools	Results
Usability and preliminary effectiveness of an app-based physical activity and education program for people with hip or knee osteoarthritis—a pilot randomized controlled trial [19]. Weber et al.; 2024	*n* = 60 (mean age = 62, 62% female); 32 in the intervention group and 28 in the control group.	App (Join2Move).	12 weeks; Physical activity (PA) (swimming or cycling), therapeutic exercise (core stability/postural function, postural orientation, lower extremity muscle strength and functional exercises), and an education program.	Participants in the control group were free to receive usual care, such as physiotherapy, which was covered by statutory health insurance funds.	12 weeks.	Primary outcomes: Usability (SUS, feedback); Physical function (KOOS); Satisfaction (ZUF-8, Join2move questionnaire); Secondary outcomes: isometric and isokinetic muscle strength (dynamometer, Biodex System 4, 30 Second Sit-to-Stand Test); ROM (analog goniometer); PA levels (IPAQ); Participation and self-management (Patient Activation Measure).	Acceptable usability, reduction in pain, no effects on physical functioning, increased PA level, reduction in isokinetic muscle strength, and no differences in all the other secondary outcomes in the comparison with the control group.
The effect of innovative smartphone application on adherence to a home-based exercise program for female older adults with knee osteoarthritis in Saudi Arabia: a randomized controlled trial [20]. Alasfour et al.; 2020	*n* = 40; 20 in the app group (intervention) (mean age = 53.65, 100% female) and 20 in the paper group (control) (mean age = 55.15, 100% female).	App (My Dear Knee).	6 weeks; Strength exercises for lower limbs following the app instructions. It was performed without the direct guidance of a physiotherapist.	Same home therapeutic exercise program for lower limbs strengthening, as the intervention group, but received as hand-outs and to be performed without the direct guidance of a physiotherapist.	3 and 6 weeks.	Primary outcomes: Adherence rate (self-reported). Secondary outcomes: Pain (ANPRS); Pain and physical function (ArWOMAC); Strength (FTSST).	In the comparison, the app group reached a significantly higher adherence and a significantly greater improvement in pain. No differences in physical function between groups at the end of week 6. No significant differences between the groups in terms of lower-limb muscle strength.
Cost-effectiveness of a blended physiotherapy intervention compared to usual physiotherapy in patients with hip and/or knee osteoarthritis: a cluster randomized controlled trial [21]. Kloek et al.; 2018	*n* = 208; 109 in the e-Exercise group (intervention) (mean age = 63.8, 67% female) and 99 in the Usual physiotherapy group (control) (mean age = 62.3, 67% female).	App (e-Exercise).	12 weeks; Five face-to-face sessions with a physiotherapist integrated with some web-based sessions, consisting of a graded activity module (walking or cycling), therapeutic exercises (for strength and stability), and information modules.	Usual physiotherapy (information, therapeutic exercise for strength and stability in face-to-face sessions under the physiotherapist’s guidance).	3 and 12 months.	Quality of life (EQ-5D-3 L); Functionality (KOOS); PA level (accelerometer); Intervention costs; Healthcare costs; Sports Costs; Informal care costs; Absenteeism costs; Presenteeism costs; Unpaid productivity costs.	No significant differences between the groups in terms of health-related quality of life, physical function, PA, primary healthcare costs, secondary healthcare costs, informal care costs, absenteeism costs, presenteeism costs and unpaid productivity costs. Intervention costs, medication costs and sports costs for the intervention group were significantly lower compared to the control group. ICER estimates analysis suggested potential dominance of TR, although with substantial statistical uncertainty.
Effectiveness of a Blended Physical Therapist Intervention in People With Hip Osteoarthritis, Knee Osteoarthritis, or Both: A Cluster-Randomized Controlled Trial [22]. Kloek, Bossen, et al.; 2018	*n* = 208; 109 in the e-Exercise group (mean age = 63.8, 67% female) and 99 in the Usual Physical Therapy group (control) (mean age = 62.3, 67% female).	App (e-Exercise).	12 weeks; Five face-to-face sessions with a physical physiotherapist and some online application-based sessions focusing on behavioral graded activity (walking or cycling), therapeutic exercises (strength and stability), and information.	Usual physical therapy (information, therapeutic exercises for strength and stability). No restrictions were given regarding the number of face-to-face sessions.	3 and 12 months.	Primary outcomes: Physical function (function in the daily living subscale of KOOS, TUG test); PA (SQUASH, accelerometer). Secondary outcomes: symptoms and functional limitations (pain, symptoms, sport/recreation function, and quality of life subscales of KOOS); Self-perceived effect, pain and tiredness (11-point NRS); Self-efficacy (ASES); Usability of website (SUS); Interpretation of website.	No significant differences between the groups were found in physical function and free-living PA. Both groups had a similar and significant improvement in terms of physical function and secondary outcomes soon after treatment and after 12 months.
Effects of a 12-Week Digital Care Program for Chronic Knee Pain on Pain, Mobility, and Surgery Risk: Randomized Controlled Trial [23]. Mecklenburg et al.; 2018	*n* = 155 (mean age = 46; 37% female); 101 in the intervention group (mean age = 46, 43% female) and 54 in the control group (mean age = 47, 26% female).	App (Hinge Health) and Bluetooth sensors.	12 weeks; Synchronous and remote sessions with a therapist; participation was completed entirely remotely with the support of a personal coach. On a weekly basis, 3 weekly sessions of sensor-guided therapeutic exercise therapy, reading education articles, logging symptoms, performing cognitive behavioral therapy, working on weight loss and tracking at least three 30-min sessions of aerobic activities.	Usual care; education providing participants with three papers related to the importance of self-care, how to deal with setbacks in knee pain, and how to manage communication and relationships when living with chronic knee pain.	12 weeks.	Primary outcomes: Pain (pain subscale of KOOS); Physical functions (KOOS-PS). Secondary outcomes: Pain (VAS, questions about pain symptoms).	In the comparison, the intervention group showed significantly better outcomes across all outcomes.
Face-to-face and telerehabilitation delivery of circuit training have similar benefits and acceptability in patients with knee osteoarthritis: a randomised trial [24]. Aily et al.; 2023	*n* = 100 (mean age 55, 60% female); 50 in the intervention group (mean age = 55, 60% female) and 50 in the control group (mean age = 53, 60% female).	Video recordings delivered via DVD, website, YouTube channel and/or videos via app (WhatsApp) videos.	14 weeks; Circuit training of therapeutic exercises delivered via video recordings, followed by periodic phone calls to motivate and instruct participants.	The same therapeutic exercise sessions in a face-to-face way under a physiotherapist’s guidance.	14 and 26 weeks.	Primary outcomes: Pain (100-mm VAS); Physical function (physical function subscale of WOMAC). Secondary outcomes: Physical function (40 m fast-paced walk test in m/s, 30 CST, stair climb test); Knee extensor strength (dynamometer); Thigh composition (tomography); Body composition (dual energy X-ray absorptiometry); Muscle architecture (ultrasound); Pain catastrophising (PCS). Adherence and acceptability.	Both groups reported similar results in terms of pain intensity, physical function, muscle strength, pain catastrophising, thigh composition, adherence, intermuscular adipose tissue and muscle architecture. Whole body composition did not change in both groups.
Development of a mobile application to improve exercise accuracy and quality of life in knee osteoarthritis patients: a randomized controlled trial [25]. Thiengwittayaporn et al.; 2023	*n* = 82; 42 in the mobile application group (intervention) (mean age = 62.2, 85% female) and in the handout group (control) (mean age = 63, 92% female).	App (Love-Your-Knee).	4 weeks; Three therapeutic strength and stretching exercises for strengthening and stretching (catch-bend-down, stretch-touch-feet and sit-stretch-hold) performed remotely and without the guidance of the physiotherapist, following the app contents.	The same therapeutic exercises as the intervention group (remotely and without the guidance of the physiotherapist), but according to handout.	4 weeks.	Participant’s ability to correctly perform the exercises; knee ROM (goniometer); Symptoms and physical function (symptoms, pain, activities of daily living, sports and recreation activities, and quality of life subscales of KOOS); Satisfaction, expectation and functional activity (KSS).	In the comparison, the intervention group reported better results in terms of exercise accuracy, activities of daily life, quality of life, ability to do sports and recreational activities, and satisfaction. No differences between groups about ROM.
Is telephysiotherapy an option for improved quality of life in patients with osteoarthritis of the knee? [26]. Odole et al.; 2014	*n* = 50 (mean age = 55.5, 48% female); 25 in the telephysiotherapy group (intervention) (mean age = 56.04, 44% female) and 25 in the clinic-based group (control) (mean age = 54.96, 52% female).	Mobile telephone.	6 weeks; Self-administered standardized therapeutic exercise programmes for people with KOA. KOA exercises monitoring and coaching were carried out about the exercise programmes via mobile telephone.	Physiotherapists administered a standardized therapeutic exercise program for KOA without monitoring or coaching via mobile telephone.	2, 4 and 6 weeks.	Quality of life (WHOQoL-Bref).	Both groups improved in physical health and psychological domains, with no significant differences in social relationships and environment domains. No differences were found between the groups in physical health, psychological, and social relationship domains.
Effect of Telerehabilitation-Based Exercise and Education on Pain, Function, Strength, Proprioception, and Psychosocial Parameters in Patients With Knee Osteoarthritis [27]. Tümtürk et al.; 2024.	*n* = 57 (mean age = 52.56, 68% female); 29 in the TR group (intervention) (mean age = 53.59, 79% female) and 28 in the paper group (control) (mean age = 51.5, 57% female).	App (WhatsApp).	8 weeks; Exercise program based on stretching, strengthening, and stabilization exercises for the muscles around the knee.	Instructional paper forms containing the same exercise program as the intervention group, education, and recommendations.	8 weeks.	Pain (VAS); Physical function (WOMAC, TUG test, FTSST, 3MBWT, mFSST); Muscle strength (dynamometer); Proprioception (inclinometer); Quality of life (EQ-5D-5L); Telemedicine Satisfaction Questionnaire; Telehealth Usability Questionnaire.	Both groups improved in terms of pain, physical function, quality of life, and muscle strength. However, the intervention group reported significantly better improvements in the aforementioned outcomes. The intervention group also reported significantly better improvements in left extremity proprioception and adherence.
Telerehabilitation consultations with a physiotherapist for chronic knee pain versus in-person consultations in Australia: the PEAK non-inferiority randomised controlled trial [28]. Hinman et al.; 2024.	*n* = 394; 190 in the TR group (intervention) (mean age = 60.5, 64% female) and 204 in the in-person group (control) (mean age = 62.2, 72% female).	App (Zoom).	12 weeks; Individualised home-based strengthening programme and physical activity plan. Education about osteoarthritis and its management.	The same treatment of the intervention group, but in a face-to-face way under a physiotherapist’s guidance.	3 and 9 months.	Pain (11-point NRS); Physical function (WOMAC); Quality of life (AQoL-6D); Physical activity levels (PASE); Arthritis Self Efficacy Scale (ASES); global changes (seven-point Likert scales); Medications and consults outside the trial (survey); Quality of the therapeutic alliance (WAI-SF); Consultation satisfaction (seven-point Likert scale); Consultation convenience (NRS); Attendance recorded by physiotherapists; Adherence (NRS); Number of strength sessions completed in the previous week; Consultation duration; Distance travelled for consultations; Adverse events; Cost-effectiveness	Both groups reported improvements for primary outcomes such as pain and function. Telerehabilitation was non-inferior for pain and function, and the number of adverse events was similar between groups.

**List of abbreviations**: 11-point NRS—Numeric Rating Scale; 30 CST—30-s Chair Stand Test; 3MBWT—3-Meter Backward Walk Test; ANPRS—Arabic Numeric Pain Rating Scale; App—Application; AQoL-6D—Assessment of Quality of Life instrument—Six Dimensions; AQoL-8D—Assessment of Quality of Life instrument—Eight Dimensions; ASES—Arthritis Self-Efficacy Scale; ArWOMAC—Arabic version of the reduced Western Ontario and McMaster Universities Osteoarthritis Index; CPM—Conditional Pain Modulation; EARS—Exercise Adherence Rating Scale; EQ-5D-3L—European Quality of Life five Dimensions, 3 Level Version; EQ-5D-5L—European Quality of Life five Dimensions, five Level Version; FSS—Fatigue Severity Scale; FTSST—Five-Times Sit-to-Stand Test; HADS—Hospital Anxiety and Depression Scale; ICERs—Incremental Cost-Effectiveness Ratios; IPAQ—International Physical Activity Questionnaire; IPAQ-SF—International Physical Activity Questionnaire Short Form; KOOS—Knee Injury and OA Outcome Score; KOOS-PS—KOOS Physical Function Shortform; KSS—Knee Society Scores; LEAS—Lower Extremity Activity Scale; mBQ—Modified Baecke Physical Activity Questionnaire; mFSST—Modified Four-Square Step Test; MSK-HQ—Arthritis Research UK Musculoskeletal Health Questionnaire; NPRS—Numerical Pain Rating Scale; PA level—Physical Activity Level; PAR-Q+—Physical Activity Readiness Questionnaire for Everyone; PASE—Physical Activity Scale for the Elderly; PCS—Pain Catastrophising Scale; PPT—Pressure Pain Threshold; QUIPA—Quality Indicators Questionnaire for Physiotherapy Management of Hip and Knee Osteoarthritis; ROM—Range of Motion; SEE—Self-Efficacy for Exercise scale; SF-12—Short-Form 12; SQUASH—Short Questionnaire to Assess Health-Enhancing Physical Activity; STEP-KOA—Stepped Exercise Program for Patients with Knee OsteoArthritis; SUS—System Usability Scale; TKS—TAMPA Kinesiophobia Scale; TSK—Tampa Scale of Kinesiophobia; TS—Temporal Summation; TUG—Timed “Up & Go” Test; VAS—Visual Analogue Scale; WAI-SF—Working Alliance Inventory Short Form; WHOQoL-Bref—World Health Organisation Quality of Life-Bref; WOMAC—Western Ontario and McMaster Universities Osteoarthritis Index.

Half of the selected studies implemented the same therapeutic exercise program for both the intervention and control groups, with the only difference being the absence of TR in the control group [20,24,25,27,28]. In contrast, four studies adopted usual care as the control condition [19,21,22,23]. In the study conducted by Odole et al., the control group followed a physiotherapist-administered standardized therapeutic exercise program for KOA without mobile phone-based monitoring or coaching [26]. In summary, control conditions ranged from an identical therapeutic exercise program as the intervention group but without TR, to usual care, to supervised in-person physiotherapy.

The included studies showed a wide variation in outcome measures. In most studies, pain related to KOA was the primary outcome, considered both as a symptom and in terms of its functional and quality of life impact. Therefore, the studies focused on pain, physical function, quality of life, PA levels, participants’ satisfaction, cost-effectiveness and adherence to the intervention as key measures of intervention success. Almost all studies (*n* = 9/10; 90%) adopted a combination of different outcomes to assess the effectiveness of the intervention [19,20,21,22,23,24,25,27,28], whereas the study by Odole et al. relied on a single quality of life outcome [26].

Seven of the selected studies included pain as an outcome [20,22,23,24,25,27,28]. Specifically, three studies used the pain subscales of the Knee Injury and Osteoarthritis Outcome Score (KOOS) [22,23,25], two studies used the Visual Analogue Scale (VAS) [24,28], two studies used the 11-point Numeric Rating Scale (11-point NRS) [22,28], while Alasfour et al. preferred the Arabic Numeric Pain Rating Scale (ANPRS) and the Arabic version of the WOMAC (ArWOMAC) for their study [20].

Physical function outcomes were the most frequently reported, appearing in nine studies [19,20,21,22,23,24,25,27,28]. Half of the studies adopted the KOOS questionnaire, either in its full version [19,21], in short-form variants [23], or by focusing on specific subscales [22,25]. The Western Ontario and McMaster Universities Osteoarthritis Index (WOMAC) questionnaire was also included as an outcome in the research conducted by Hinman et al. [28] and Tümtürk et al. [27], and for the physical function subscale by Aily et al. [24]. Additional functional performance tests included the Five-Times Sit-to-Stand Test (FTSST) [20,27], Timed “Up & Go” (TUG) test [22,27], Modified Four-Square Step Test (mFSST) [27], 3-Meter Backward Walk Test (3MBWT) [27], 40 m fast-paced walk test in m/s [27], 30 s chair stand test (30 CST) [27] and stair climb test [27]. Only one study included proprioception as a measure to assess the efficacy of treatment [27].

Muscle strength was evaluated in several studies using dynamometers [19,23,27]. Additionally, two studies included a range of motion (ROM) measured with a goniometer [19,25].

Quality of life was assessed exclusively using the World Health Organisation Quality of Life-Bref (WHOQoL-Bref) in the study carried out by Odole et al. [26]. Other studies evaluated quality of life evaluation alongside additional outcomes using EuroQol-5 Dimensions, Three Levels (EQ-5D-3L) [21], EuroQol-5 Dimensions, Five Levels (EQ-5D-5L) [27], or Assessment of Quality of Life instrument—Six Dimensions (AQoL-6D) [28].

PA levels were assessed using both objective tools, such as accelerometers [21,22], and self-report questionnaires, including the International Physical Activity Questionnaire (IPAQ) [19], Physical Activity Scale for the Elderly (PASE) [28], and Short Questionnaire to Assess Health-Enhancing Physical Activity (SQUASH) [22].

Participants’ satisfaction was evaluated in four studies [19,25,27,28]. Two studies assessed the cost-effectiveness of TR, focusing on expenses related to healthcare, sports, informal care, absenteeism, presenteeism, and unpaid productivity [21] or considering additional costs per quality-adjusted life-years, intervention costs, knee-related health-care use outside the trial, as well as participant time and travel costs [28].

Additional outcomes included treatment adherence [19,20,24,28], app usability [19,22,27], body composition and muscle architecture [24], pain catastrophizing [24], time spent and distance travelled for consultations [28], and other self-reported measures (e.g., self-efficacy, self-perceived effects, tiredness, adverse events) [22,28].

Four studies [19,20,23,27] reported significant improvements in pain in the intervention group compared to the control group. In contrast, two studies [24,28] found no statistically significant between-group differences in pain reduction.

Five studies [19,21,22,24,28] found no significant differences between the intervention and control groups for physical function outcomes, although significant within-group improvements were observed. One study demonstrated significantly superior functional outcomes in the intervention group [27]. Thiengwittayaporn et al. observed improvements in functional outcomes such as KOOS and Knee Society Score (KSS), in both groups, with greater improvements in the intervention group [25].

Regarding walking speed, sit-to-stand test, stair climb test, muscle torque, and muscle architecture, Aily et al. found improvements in both groups, with minimal between-group differences [24]. Moreover, Thiengwittayaporn et al. reported no significant improvements in knee ROM in either group [25].

Alasfour et al. found no significant between-group differences in muscle strength improvement, although both groups improved over time [20]. Weber et al. observed a significant between-groups difference in isokinetic strength (flexion 60° total work), in favor of the intervention group [19]. Moreover, Tumturk et al. found greater hamstring strength in the control group, while the intervention group demonstrated better proprioception and functional performance, particularly in the FTSST [27].

Regarding quality of life, Odole et al. reported improvements in the physical health and psychological domains in both groups, with no differences in social relationships and environmental domains [26]. Kloek et al. found no significant differences between groups in terms of health-related quality of life [21], whereas Tumturk et al. and Thiengwittayaporn et al. reported better quality of life improvements for the intervention group [25,27].

Weber et al. and Hinman et al. highlighted increased PA levels in the intervention group [19,28], while other studies found no significant differences between the groups for this outcome [21,22,28]. Three studies [20,27,28] reported significantly higher adherence rates in the intervention group compared to the control, whereas Aily et al. observed a high adherence rate (>95%) in both groups [24].

Furthermore, the selected studies reported that participants in the intervention group experienced higher satisfaction [25,28], improved daily activities, improved sports performance, higher expectations, and greater accuracy in executing the prescribed exercises [25].

Finally, Hinman et al. reported that TR was cost-saving, particularly for participants living outside the largest cities [28]. Kloek et al. conducted a formal cost-effectiveness analysis including ICER estimates [21]. Their study found that intervention costs, medication costs, and sports costs were significantly lower for the intervention group compared to the control group, although no significant differences were reported regarding primary healthcare costs, secondary healthcare costs, informal care costs, absenteeism costs, presenteeism costs, and unpaid productivity costs. However, the results from ICER estimates showed substantial statistical uncertainty, thus highlighting that this potential greater cost-effectiveness of TR should be interpreted with caution.

### 3.4. Assessment of Methodology and Quality of the Studies

The methodological quality of the included studies was assessed using the Modified Jadad Score, as shown in Table 2. In this systematic review, six studies were identified as high quality (scores > 4) [20,22,23,24,25,28], while four studies were categorized as moderate quality (scores 3 to 4) [19,21,26,27]. No studies were classified as low quality (scores < 3). Overall, the predominantly moderate to high quality of the included studies enhances the reliability of these findings. Detailed results are presented in Table 2.

### 3.5. Evaluation of Risk of Bias

**Figure 2:**
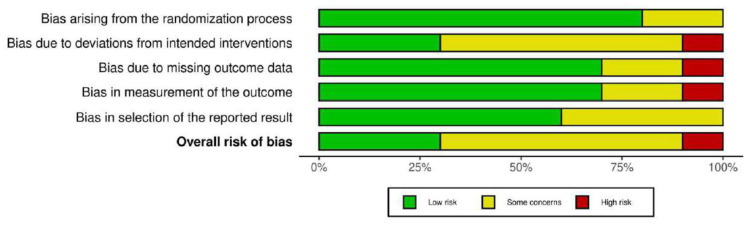
Risk of bias graph.

The risk of bias was evaluated, and it was summarized in Figure 2. The overall level of risk of bias in all the studies selected in this systematic review showed some concerns regarding the randomization process (selection bias), deviations from the intended intervention (performance bias), blinding of the outcome assessment (detection bias), incomplete measurement of the outcome data (attrition bias), and selective reporting of the results (reporting bias).

**Figure 3:**
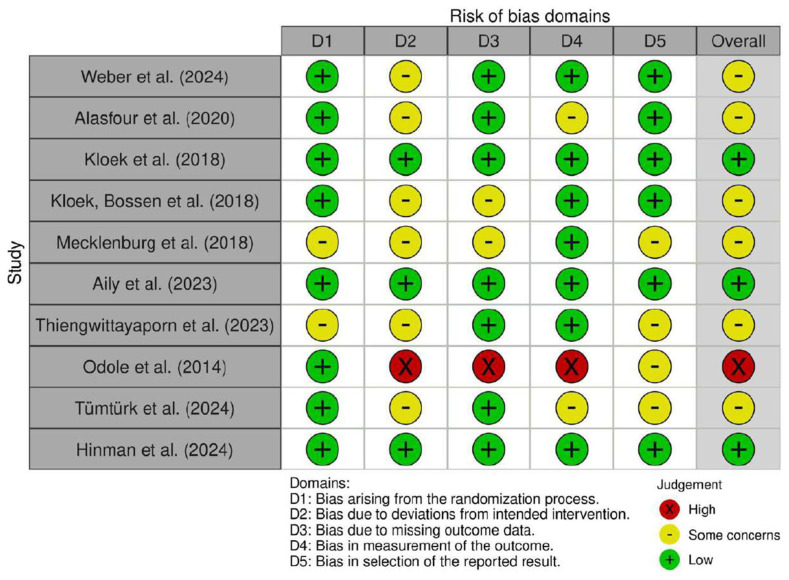
Traffic-light plot of risk of bias assessment across included randomized controlled trials [19,20,21,22,23,24,25,26,27,28].

A summary of the risk of bias is shown in Figure 3. It reveals that three of the ten studies presented an overall low risk of bias, and six presented some concerns, especially due to deviations from the intended intervention. Conversely, only one of the ten studies showed a high risk of bias due to deviations from the intended intervention, missing outcome data and possible bias in the measurement of the outcomes.

### 3.6. Heterogeneity

Potential sources of heterogeneity were qualitatively assessed based on study design, intervention, treatment, outcomes, technological platforms and outcome measures. A considerable level of clinical and methodological heterogeneity was observed across the selected studies. Given this marked variability, a meta-analysis could not be performed.

## 4. Discussion

Telemedicine is a rapidly expanding field in healthcare, although it is not a recent discovery. Since the 1970s, it has represented a potential means of connecting hospitals and remote areas, thereby attracting sustained interest from the scientific community [29]. However, it is only over the last decade that the research in this field grown exponentially, largely due to rapid technological progress applied to medicine. Most notably, the COVID-19 pandemic provided a decisive impetus for the widespread adoption of telemedicine. The dramatic spread of COVID-19 and the restrictions imposed to limit its dissemination profoundly disrupted daily life worldwide [30,31,32], including the traditional organization of healthcare systems [33]. The management of common musculoskeletal pathologies has also been impacted by the pandemic [34], prompting healthcare professionals to adopt alternative models of care and treatment delivery [35]. Moreover, the use of telemedicine, particularly for musculoskeletal disorders, has remained substantial even beyond the COVID-19 emergency [36,37], highlighting the importance of the use of technology in rehabilitation. In this context, KOA was no exception and became a relevant model for healthcare reorganization. Endstrasser et al. demonstrated that the COVID-19 lockdown had a significant impact on pain, joint function, physical function, and PA in people with end-stage hip osteoarthritis and KOA [38], while Tenti et al. showed how traditional injection treatments were affected during that dramatic period [39].

Consequently, telehealth solutions for KOA have become increasingly urgent and useful. Faced with an increase in telemedicine therapeutic options for people suffering from KOA [40], comprehensive evidence regarding their overall benefits remains limited, in particular with respect to exercise-based TR, which is undoubtedly a treatment with multiple positive implications.

It is important to note that the included studies in this systematic review demonstrated considerable heterogeneity in terms of intervention types, technological platforms, treatment intensity and duration, assessed outcomes, and the conditions of the control group. This variability reflects the diversity of current TR approaches for KOA and necessitates cautious interpretation of findings. Differences in exercise type, dosage, duration, technological tools, and levels of supervision may contribute to variable clinical response and may have influenced both the magnitude and consistency of the observed effects. Therefore, the overall conclusions should be interpreted with caution, and the findings cannot be uniformly generalized across all TR settings.

Overall, this systematic review suggests that exercise-based TR for KOA seems to guarantee many advantages. Firstly, pain has been largely investigated in seven of the selected studies. Compared with traditional rehabilitation, TR was better in relieving pain according to all the different scales used in four studies [19,20,23,27], while it was found to be non-inferior in the remaining three [22,24,28]. These findings are consistent with current evidence. A recent systematic review and meta-analysis by Wang et al. reported moderate-quality evidence indicating that internet-based telehealth programs for people suffering from hip and KOA produced superior pain relief (SMD −0.27, 95% CI −0.34 to −0.19, *p* < 0.001) and improved pain-related self-efficacy (SMD 0.21, 95% CI 0.08–0.34, *p* < 0.001) compared with conventional rehabilitation [41].

Physical function is another key domain impaired by KOA. Our results suggest that TR may be more effective than traditional approaches for certain functional outcomes, including KOOS, WOMAC, TUG test, FTSST, 3MBWT, mFSST, and KSS, with differences between individual studies relating to the specific scales used [20,23,25,27]. However, these findings deserve careful consideration. In fact, a recent meta-analysis by Chen et al. showed that technology-supported exercise programs for KOA and chronic knee pain were associated with significant pain reduction but not with consistent improvements in physical function [10]. This discrepancy may be explained by the wide range of assessment tools used to evaluate physical function. Therefore, the functional scales predominantly based on pain assessment, such as KOOS and WOMAC, tend to record good results, comparable to those obtained for pain. On the contrary, evaluation scales and outcome measures focused on more specific aspects of knee functionality may yield heterogeneous or apparently conflicting results.

In fact, Thiengwittayaporn et al. reported improvements in physical function based on KOOS and KSS, while observing no between-group differences in knee ROM [25]. On the other hand, Tümtürk et al. identified significantly greater improvement in the TR group in terms of muscle strength, measured using a dynamometer, and proprioception, measured with an inclinometer [27]. These findings suggest that future research incorporating more objective and precise outcome measures may better capture the functional benefits of TR.

Nevertheless, heterogeneity in between-group functional outcomes was evident. In fact, five studies [19,21,22,24,28] found no significant differences between the intervention and control groups for physical function outcomes. This heterogeneity could be partially explained by differences in study design. In these trials, both groups achieved similar and clinically meaningful improvements, suggesting that TR is not necessarily superior, but rather comparable to high-quality conventional rehabilitation when treatment supervision and therapeutic focus are equivalent. In particular, the differences in improvements between TR groups and control groups were smaller when the control group underwent supervised traditional physiotherapy [19,21,22,24,28] compared with studies in which the control group received lower levels of supervision [20,23,25,27]. Furthermore, interventions focusing on specific strengthening exercises with close attention to execution [20,25,27] appeared to favour TR, whereas more complex, multicomponent programmes incorporating behavioral and educational elements [21,22,28] often produced similar outcomes across groups.

Quality of life represents another clinically important outcome and was assessed in several included studies. Although different measurement tools were used, TR appeared to be equally effective or even superior to face-to-face rehabilitation in improving quality of life among people with KOA, as reported by Tümtürk et al. [27]. These findings are supported by a systematic review by Schäfer et al., which highlighted the efficacy of eHealth interventions compared with no intervention or traditional rehabilitation in improving health-related quality of life [42]. However, these same authors defined the differences in favour of TR as small and inconsistent; therefore, further studies testing single and standardized quality of life outcome measures are necessary, also because these outcomes are self-reported by participants.

Improvement in PA levels represents another important benefit of exercise-based TR. Among the included studies, TR was at least as effective as traditional therapeutic approaches based on physical exercise in increasing the PA levels, regardless of the used evaluation tool. Increases in PA likely reflect improvements in knee pain and physical function and may also contribute to slowing disease progression, particularly when PA is performed according to dedicated mobile apps [43]. The mutual relationship between PA and therapeutic exercise in osteoarthritis is well established, and current KOA guidelines identify PA as a cornerstone of patient education management [44]. The advent of new technologies has made it possible to respond to a growing demand for knowledge and practice of rehabilitation by people. Making it more easily usable and practicable independently in the TR modality is the key to leading them to more adequate and more protective lifestyles with respect to the development of chronic pathologies, such as osteoarthritis [45].

Treatment adherence [20,27,28] and participants’ satisfaction [25,28] were also frequently reported and are closely interrelated outcomes. These measures were significantly higher in TR groups compared with controls, highlighting the potential of TR to enhance patient engagement. Participants’ adherence is particularly critical in rehabilitation, which requires active involvement and constant commitment. TR enables remote guidance, real-time feedback and continuous monitoring, often within a patient’s home environment [46,47,48]. Moreover, reduced transport costs and the possibility of performing rehabilitation at home make TR more feasible and attractive. Rossi et al. reported high patient satisfaction and adherence in users of a smartphone-based care management platform following robotic total knee arthroplasty, although some limitations related to usability were also identified [49]. These findings emphasise the challenge of ensuring equitable access to digital technologies and optimising usability across diverse populations.

Finally, two of the included studies reported cost-related outcomes in favour of TR [21,28]. Regardless of the specific outcomes investigated, these findings suggest that TR may offer economic advantages for both patients and healthcare organizations, although the overall evidence remains limited. Kloek et al. conducted a cost-effectiveness analysis including ICER estimates and reported potential dominance of e-Exercise over usual physiotherapy for Quality Adjusted Life Years (QALYs) and physical functioning, although with substantial statistical uncertainty [21]. A meta-analysis by Molina-Garcia et al. showed that TR was USD 89.55 (95% CI 4.6 to 174.5) cheaper per individual than conventional treatments for musculoskeletal disorders [50]. Moreover, TR saved a mean of 4 h and 27 min over the duration of the rehabilitation, ranging from 3 h and 50 min to 5 h and 18 min. Similarly, Buvik et al. found that telemedicine costs USD 73 less per person than traditional consultations at the outpatient services, and also that TR saved an average of 7 h and 40 min travel time in comparison with face-to-face rehabilitation [51]. Overall, results suggest that exercise-based TR may reduce specific cost components, such as intervention and medication costs. However, only one study included ICER estimates in its analysis and reported a substantial statistical uncertainty. Moreover, the limited number of economic evaluations currently available prevents drawing definitive conclusions with regard to the overall cost-effectiveness of TR. Thus, further methodologically rigorous studies are needed to confirm these preliminary findings.

Although evidence in this area is still emerging, the results of this systematic review are encouraging.

In conclusion, this systematic review indicates that TR offers multiple advantages for people with KOA. Nevertheless, several barriers to widespread implementation remain, including unequal access to technology, variable digital literacy, and the need for enhanced telemedicine training among healthcare professionals. Nevertheless, TR represents a significant opportunity for patients and healthcare systems all over the world. In the future, research should focus on developing and validating standardised TR protocols, facilitating their integration into international clinical guidelines, as has already occurred in other fields of rehabilitation [52,53].

To move towards defining standardized protocols and addressing current gaps in the scientific literature, we are launching a multicenter randomized controlled clinical trial to evaluate the efficacy and safety of a novel digital therapy for KOA. This trial involves five Italian Universities, namely University of Salento, Sapienza University of Rome, Campus Bio Medico University of Rome, University of Foggia and “G. D’Annunzio” University of Chieti and Pescara. This study protocol will evaluate Symptomatic Digital Drugs for Osteoarthritis (SyDiDOa), a web app developed for individuals suffering from KOA. The aim of SyDiDOa is to provide a personalized, technology-supported rehabilitation program designed to reduce pain and improve function. This project may help address several limitations currently reported in scientific literature, such as the lack of technical standardization and the heterogeneity of intervention protocols, while also improving the personalization of exercise-based TR programs.

The study will include three treatment arms: conventional conservative therapy, SyDiDOa therapy, and a combination of both, with follow-up assessments at 1, 3, and 6 months. SyDiDOa incorporates artificial intelligence and real-time video motion tracking to guide patients through therapeutic exercises and provide biofeedback to guarantee correct execution. Moreover, it allows clinicians to personalize programs based on individual clinical and anthropometric. These therapeutic exercises focus on strengthening and proprioception for the anterior and posterior muscles of the thigh and leg. They are the same as those used in conventional conservative therapy of the study, but are performed under the guidance of the web app in biofeedback mode, always under the supervision of an experienced physiotherapist. The main objective is to determine whether digital therapy using SyDiDOa is at least as effective and safe as conventional rehabilitation while offering greater accessibility and personalization. If successful, this trial may provide significant evidence supporting the integration of TR into KOA management, promoting a more tailored and sustainable model of care. The SyDiDOa project has been registered among the projects funded by PRIN (Research Projects Of Relevant National Interest—m_pi.AOODGRIC.REGISTRO_PRIN2022.0002190.30-03-2022—Next Generation EU, Mission 4, Component 1 CUP B53D23020170006) and received a sum of EUD 334.163 (Prot. 2022CCFXEX) to develop the above-mentioned app and evaluate its effectiveness.

This systematic review has several limitations. The wide range of technologies used was quite large, including several different apps, video recordings, and auxiliary tools. However, the purpose of this study was to evaluate TR from a general perspective, as long as it is always based on therapeutic exercise. Another limitation of this systematic review is the heterogeneous nature of the interventions, although they are all related to therapeutic exercise. Because of this limitation, a meta-analysis could not be performed. Moreover, the substantial heterogeneity across interventions and outcome measures may have reduced the comparability of results and limited the robustness of the conclusions. Despite the issue of heterogeneity, it must be noted that one of the advantages of TR is its inherent ability to tailor rehabilitation therapy based on the person’s motor skills and specific pathological characteristics associated with KOA. An additional limitation of this systematic review concerns the relatively small number of included studies. However, this finding should be interpreted cautiously. This study prioritized the robustness of the evidence over the quantity of available studies, including only RCTs with adequate methodological quality, as assessed by the Modified Jadad Score and RoB 2. In this context, the limited number of eligible studies may also be considered a relevant result in itself, highlighting a current gap in the literature regarding high-quality exercise-based TR interventions for KOA. Moreover, the decision to include only studies published in the last 10 years may be a weakness, as earlier TR trials may have been excluded, potentially introducing selection bias. However, this decision was made to focus on interventions based on contemporary digital technologies and to highlight the most recent evidence on a topic that has developed significantly in the last decade. Further limitations of this review include intervention fidelity and comorbidities, which were not systematically assessed, as most included studies did not provide sufficiently detailed or standardized information on program adherence or participants’ comorbidities. Finally, exclusion criteria, such as language or the type of intervention, may have limited the number of papers retrieved.

On the other hand, this systematic review contributes to addressing a gap in the scientific literature by providing a comprehensive overview of all the benefits of TR, systematically reporting the wide range of outcome measures in a thorough and extensive manner. Furthermore, based on the risk-of-bias assessment conducted in this study, only one of the selected studies was found to present a high risk of bias, highlighting the overall high methodological quality and reliability of this systematic review.

## 5. Conclusions

Regardless of the technological modality used, TR provides numerous beneficial effects for people with KOA. Notably, the studies included in this systematic review were qualitatively assessed using the Modified Jadad Score, indicating a generally moderate to high methodological quality. Overall, it emerges that TR was associated with pain relief, improvements in physical function and PA levels, and enhancements in quality of life. However, some studies did not demonstrate TR’s superiority over control groups in pain relief, highlighting the need for cautious interpretation of these findings. Preliminary evidence suggests potential cost-related advantages for specific cost components; however, the available evidence on cost-effectiveness remains limited and uncertain. Due to the heterogeneity of the included studies and the limitations of this review, further high-quality studies are needed to better explore and clarify the potential of this new rehabilitation approach, strengthen the scientific evidence supporting it, and facilitate its integration into international guidelines for osteoarthritis.

## Figures and Tables

**Table 2:** The modified version of Jadad quality scores for the selected studies.

Authors	Was the Treatment Randomly Allocated?	Was the Randomization Procedure Described and Was Appropriate?	Was There a Description of Withdrawals and Dropout?	Was There a Clear Description of the Inclusion/Exclusion Criteria?	Was the Methods of Statistical Analysis Described?	Jadad Score (0–5)
Weber et al.; 2024 [19]	Yes	Yes	No	No	Yes	3
Alasfour et al.; 2020 [20]	Yes	Yes	Yes	Yes	Yes	5
Kloek et al.; 2018 [21]	Yes	No	Yes	Yes	Yes	4
Kloek, Bossen et al.; 2018 [22]	Yes	Yes	Yes	Yes	Yes	5
Mecklenburg et al.; 2018 [23]	Yes	Yes	Yes	Yes	Yes	5
Aily et al.; 2023 [24]	Yes	Yes	Yes	Yes	Yes	5
Thiengwittayaporn et al.; 2023 [25]	Yes	Yes	Yes	Yes	Yes	5
Odole et al.; 2014 [26]	Yes	Yes	No	Yes	Yes	4
Tümtürk et al.; 2024 [27]	Yes	Yes	No	Yes	Yes	4
Hinman et al.; 2024 [28]	Yes	Yes	Yes	Yes	Yes	5

## Data Availability

Data are available by the corresponding author upon reasonable request.

## Abbreviations

The following abbreviations are used in this manuscript:

KOA	Knee Osteoarthritis
TR	Telerehabilitation
SyDiDOa	Symptomatic Digital Drugs for Osteoarthritis
PA	Physical Activity
PRISMA	Preferred Reporting Items for Systematic Reviews and Meta-Analysis
